# A phosphodiesterase 5 inhibitor, tadalafil, suppresses stromal predominance and inflammation in a rat model of nonbacterial prostatitis

**DOI:** 10.1186/s12894-019-0525-x

**Published:** 2019-10-23

**Authors:** Mikio Sugimoto, Xia Zhang, Nobufumi Ueda, Hiroyuki Tsunemori, Rikiya Taoka, Yusi Hayashida, Hiromi Hirama, Yasuyuki Miyauchi, Yuki Matsuoka, Hirohito Naito, Yu Osaki, Yosiyuki Kekehi

**Affiliations:** Department of Urology, Kagawa University Faculty of Medicine, 1750-1 Ikenobe, Miki-cho, Kita-gun, Kagawa, 761-0793 Japan

**Keywords:** Benign prostatic hyperplasia, Inflammatory cytokines, Lower urinary tract symptoms, Nonbacterial prostatitis, Tadalafil

## Abstract

**Background:**

Chronic inflammation is thought to be a major causative factor for the development of benign prostatic hyperplasia (BPH) and lower urinary tract symptoms (LUTS). Tadalafil, a phosphodiesterase type 5 inhibitor (PDE5-I), which has been used for the treatment of BPH-LUTS in daily practice, is known to act at several urinary organs. In this study, focused on the prostate, we examined the effect of tadalafil on the pathological changes and inflammatory factors in a rat nonbacterial prostatitis (NBP) model.

**Methods:**

Forty ten-month-old male Wistar rats were divided into nonbacterial prostatitis (NBP), NBP with tadalafil treatment (NBP-tadalafil), control, and control treated with tadalafil (control-tadalafil) groups (*n* = 10 per group). The NBP and NBP-tadalafil groups were castrated and then received daily subcutaneous 17β-estradiol for 30 days. The control-tadalafil and NBP-tadalafil groups were administered daily oral tadalafil for 30 days. All rats were then sacrificed and pathological changes and inflammatory factors were assessed in the prostatic tissues.

**Results:**

In the NBP group, the stroma-to-epithelium (S/E) ratio in the ventral prostate was significantly higher than in the control group (*P* < 0.001). In the NBP-tadalafil group, the S/E ratio was significantly lower than in the NBP group (*P* < 0.001). The macrophage levels and the extent of T-cell infiltration in the NBP-tadalafil group were significantly lower than in the NBP group (*P* < 0.005; *P* < 0.001, respectively). Compared with the NBP group, tissue concentrations of inflammatory cytokines, such as tumor necrosis factor-α, interleukin-8, and interleukin-1β, were significantly downregulated in the NBP-tadalafil group (*P* < 0.01; *P* < 0.05; *P* < 0.005, respectively).

**Conclusions:**

Tadalafil suppressed stromal predominance and showed anti-inflammatory effects in a rat NBP model in association with downregulation of inflammatory cytokines.

## Background

Lower urinary tract symptoms (LUTS) associated with benign prostatic hyperplasia (BPH) is common in aging males worldwide. Because LUTS/BPH often interferes with activities of daily living, many patients seek definitive treatment to improve their quality of life [1–3].

The etiology of LUTS is highly complex and is likely to be multifactorial, and several processes may contribute to the development of LUTS in BPH. Chronic inflammation in the prostate may contribute to prostate growth, tissue damage, and prostate enlargement [4]. The Medical Therapies of Prostate Symptoms (MTOPS) study, the largest study to examine chronic prostatic inflammation, suggests that the risk of serious BPH-related outcomes, including acute urinary retention and the need for BPH-related surgery, is increased. Therefore it is important to study the action of clinically used drugs on prostatic inflammation.

Phosphodiesterase type 5 inhibitors (PDE5-Is), such as tadalafil, are effective for the treatment of LUTS/BPH [5, 6], and have been acknowledged in the recent guidelines published by the Japanese Urological Association [7] and the European Association of Urology [8], with Level 1 evidence. Several LUT tissues—prostate, urethra, and bladder, as well as their vasculatures—are thought to be potential targets for tadalafil [9]. There are several reports focused on prostate inflammation regarding the mechanism of tadalafil in several animal models. Single or repeated dosing with tadalafil improves prostate hypoxia in spontaneously hypertensive rats [10]. In the prostate of rabbits fed a high-fat diet, chronic tadalafil treatment produces inhibition of inflammation, fibrosis, and hypoxia [11]. More recently, repeated treatment with tadalafil was found to suppress tactile allodynia in rat non-bacterial prostatitis induced by 17β-estradiol treatment combined with castration [12]. Although inflammatory changes, including histopathological changes and increases in several cytokine/chemokine levels, were demonstrated in this model in the absence of tadalafil treatment, whether tadalafil affects these kinds of pathological conditions in this hormone/castration-induced prostatitis model was not investigated [12].

This study aimed to elucidate the effect of the repeated administration of tadalafil on the inflammatory changes in a rat model of hormone/castration-induced prostatitis as a model of nonbacterial prostatitis (NBP) as indicated by changes in prostate histology and inflammatory factors.

## Methods

### Animals

Forty adult male 40-week-old Wistar rats (580–720 g) were purchased from Charles River Laboratories (Yokohama, Japan). Rats were housed in a climate-controlled room and allowed free access to food and water according to the Guide for the Care and Use of Laboratory Animals of Kagawa University.

### Preparation of tadalafil

Tadalafil (Cayman Chemical; Ann Arbor, MI, USA) was suspended in 0.5% methylcellulose solution (Wako, Japan) to yield a concentration of 2 mg/ml for oral administration.

### Experimental design

The 40 rats were numbered and randomly divided into 4 groups of 10 each by means of a random digits table, i.e., NBP, NBP with tadalafil treatment (NPB-tadalafil), control, and control treated with tadalafil (control-tadalafil) groups. NBP was induced according to the method of Kamijo et al. [13]. Briefly, the rats in the NBP and NBP-tadalafil groups underwent castration and then were injected subcutaneously with a daily dose of 0.25 mg/kg 17β-estradiol in 0.1 ml sesame oil for 30 days. Meanwhile, the control-tadalafil and NBP-tadalafil groups received once-daily oral tadalafil at a dose 5 ml/kg. After 30 days of tadalafil treatment, the rats were deeply anesthetized with an intraperitoneal injection of sodium pentobarbital at 200 mg/kg. Ventral prostate tissue was obtained from all rats. Some tissue samples were stored at − 80 °C for cytokine assay and the remaining tissue was fixed in 10% neutral buffered formalin and embedded in paraffin, after which 3–4 μm serial sections were made and examined histologically.

### Immunohistochemical staining

After deparaffinization, immunohistochemical staining was performed using the avidin-biotin-peroxidase complex method (SAB-PO Kit; Vector Laboratories Inc., Burlingame, CA, USA) and microwave antigen retrieval. The primary antibodies were antimacrophage rat monoclonal antibody ab56297 (Abcam, Cambridge, UK) and anti-CD3 rabbit monoclonal antibody ab16669 (Abcam). The anti-CD3 antibody was an anti-rat T-cell antibody used to identify infiltrating T-cells. The macrophages and T-cells in the sections were counted in five randomly selected fields under 400× magnification and the average was calculated.

### Evaluation of stroma-to-epithelium ratio

To evaluate the stroma-to-epithelium (S/E) ratio in the rat ventral prostate, Masson trichrome staining was performed and a computerized image-analysis system was used, as previously reported [14]. The slides were photographed using a BX51 light microscope (Olympus, Tokyo, Japan) and then analyzed with WinRoof image-analysis software (Ver. 3.6, Mitani Corporation, Japan).

### Determination of cytokines

The levels of cytokines in the supernatant of the prostate homogenate was measured as described previously [14]. Tumor necrosis factor-α (TNF-α) and interleukin-1β (IL-1β) enzyme-linked immunosorbent assay kits from R&D Systems (Minneapolis, MN, USA), and an interleukin-8 (IL-8) kit from LifeSpan BioSciences (Seattle, WA, USA) were used for the assays. Each assay was performed three times.

### Statistical analysis

Data analysis was performed using IBM SPSS software (Ver. 20; IBM, Armonk, NY, USA). The number of macrophages and T-cells, the S/E ratio in the rat prostate, and the tissue concentration of cytokines were all presented as the mean ± SD. One-way analysis of variance (ANOVA) followed by Tukey’s HSD test was used for comparison of groups. A result was considered statistically significant when the *P*-value was ≤0.05.

## Results

### Effect of tadalafil on rat body and prostate weights

After 30 days of treatment, the mean rat body weights (BW) and prostate weights (PW) in the NBP and NBP-tadalafil groups were significantly lower than in the control group (BW: *P* < 0.001, *P* < 0.001; PW: *P* < 0.001, *P* < 0.001, respectively). The mean PW and BW were not significantly different between the control and control-tadalafil groups. Toxicity associated with tadalafil treatment was not apparent throughout the study (Table 1).

**Table 1:** Body and prostate weight in NBP, NBP-tadalafil, control and control-tadalafil groups

		Mean ± SD (g)	
Group	No. Rats	Body weight	Prostate weight
NBP	10	561.9 ± 32.4**	0.45 ± 0.11**
NBP-tadalafil	10	581.4 ± 30.9**	0.65 ± 0.14**
Control	9	730.1 ± 52.4	1.34 ± 0.36
Control-tadalafil	10	694.8 ± 27.3	1.18 ± 0.43

*NBP* nonbacterial prostatitis, *NBP-tadalafil* NBP with tadalafil treatment, *Control-tadalafil* Control with tadalafil treatment. Significant differences compared with control group, ** *P* < 0.01

### Effect of tadalafil on histopathological features in the prostate

**Fig. 1:**
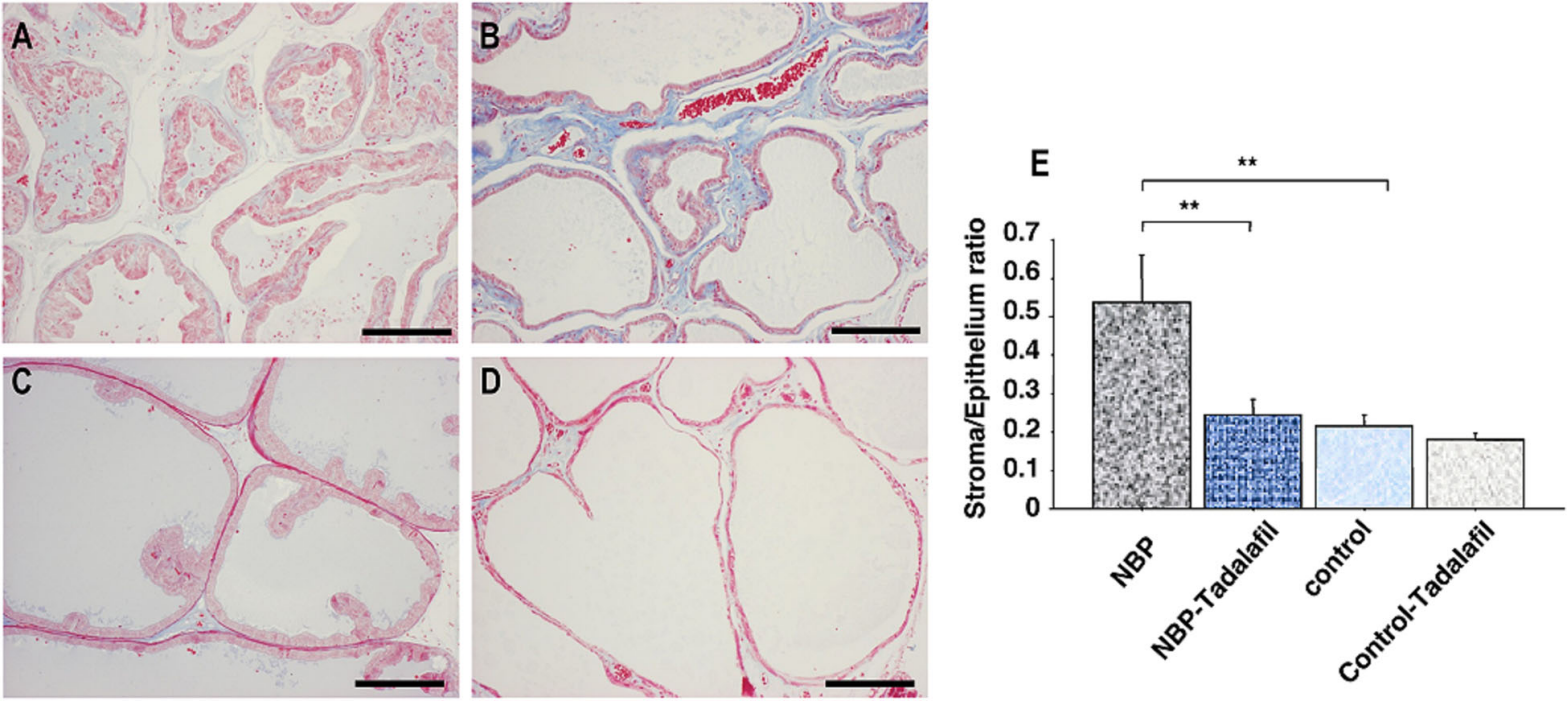
Masson trichrome staining of prostate tissue sections in NBP (**a**), NPB-tadalafil (**b**), control (**c**), and control-tadalafil (**d**) groups. Scale bars represent 100 μm. Effect of tadalafil on mean stroma-to-epithelium ratio in the prostate of NBP and non-NBP rats (**e**). Each bar represents the mean ± SD. * *P* < 0.05, ** *P* < 0.01

**Fig. 2:**
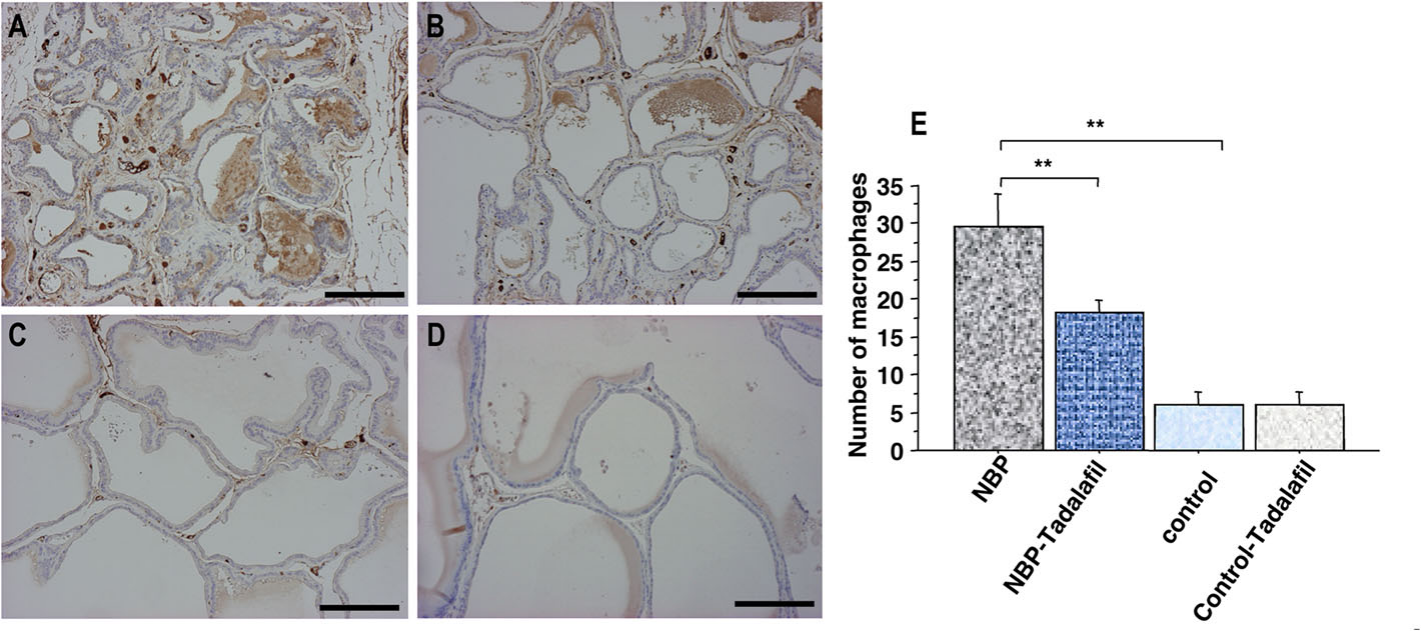
Immunohistochemical staining of paraffin-embedded prostate tissue sections with anti-macrophage antibody in NBP (**a**), NPB-tadalafil (**b**), control (**c**), and control-tadalafil (**d**) groups. Scale bars represent 100 μm. Effect of tadalafil on macrophage infiltration in the prostate of NBP and non-NBP rats (**e**). Each bar represents the mean ± SD. * *P* < 0.05, ** *P* < 0.01

**Fig. 3:**
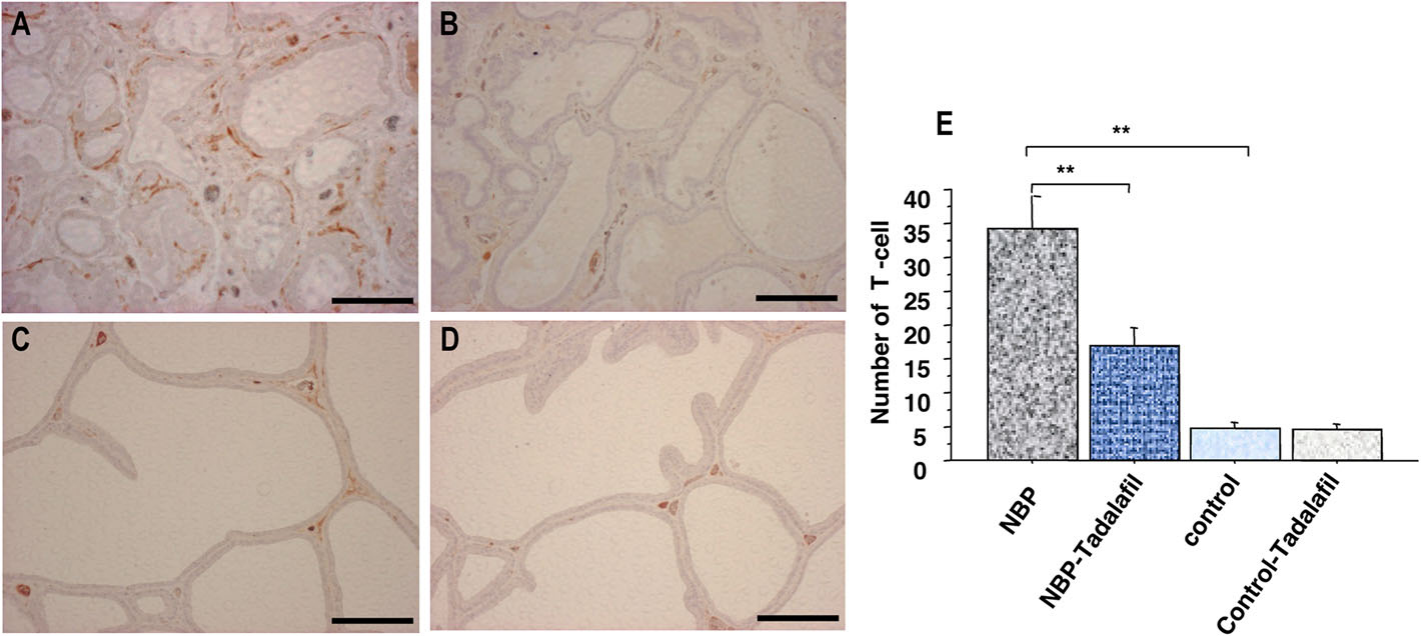
Immunohistochemical staining of paraffin-embedded prostate tissue sections with anti-CD3 antibody in NBP (**a**), NPB-tadalafil (**b**), control (**c**), and control-tadalafil (**d**) groups. Scale bars represent 100 μm. Effect of tadalafil on T-cell infiltration in the prostate of NBP and non-NBP groups (**e**). Each bar represents the mean ± SD. * *P* < 0.05, ** *P* < 0.01

The S/E ratios in NBP rats were higher than in the non-prostatitis control rats. The administration of tadalafil significantly suppressed stromal predominance (*P* < 0.001; Fig. 1). High macrophage levels and extent of T-cell infiltration surrounding the gland were observed in rats with prostatitis. The numbers of macrophages and T-cells in the ventral prostate of control rats with prostatitis were greater than in non-prostatitis control rats (*P* < 0.005, *P* < 0.001, respectively; Figs. 2 and 3). Tadalafil treatment resulted in significantly reduced macrophage and T-cell infiltration in the NBP rats (*P* < 0.001, *P* < 0.001, respectively; Figs. 2 and 3), but had no effect in non-prostatitis rats.

### Effect of tadalafil on proinflammatory cytokine levels

**Fig. 4:**
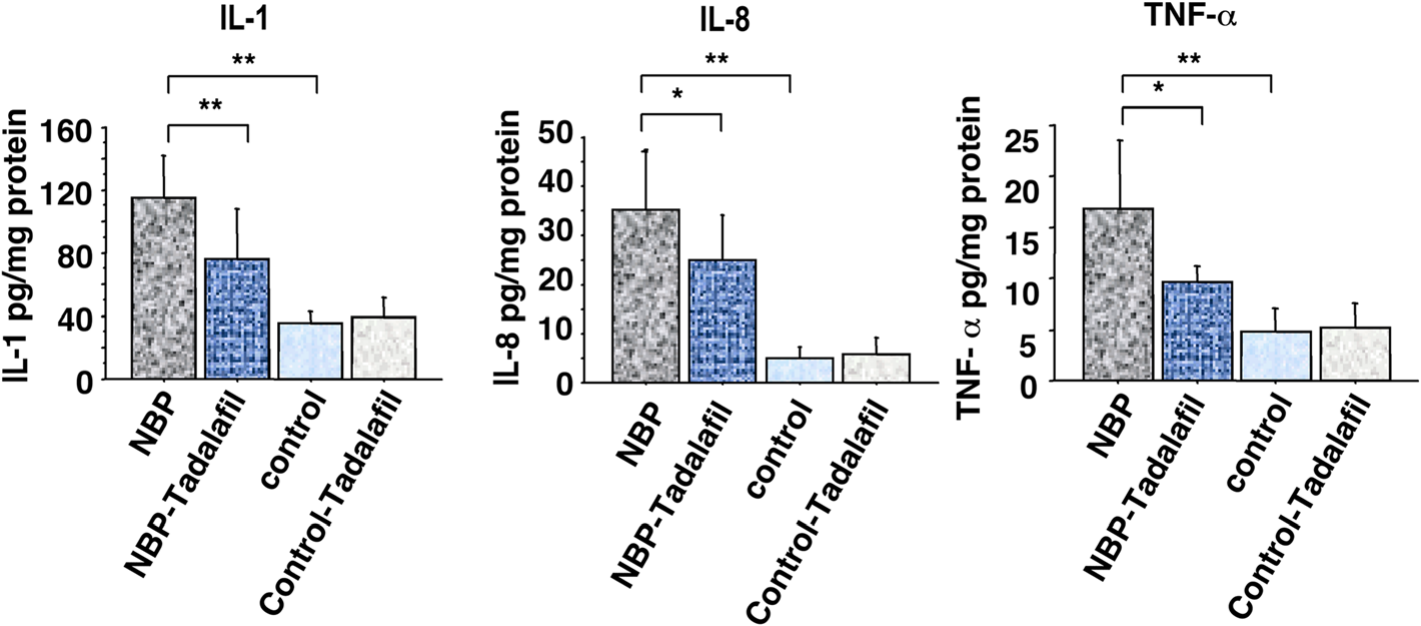
Effect of tadalafil on IL-1β, IL-8, and TNF-α levels in the prostate of NBP and non-NBP rats. Each assay was performed three times and each bar represents the mean ± SD (*n* = 10). * *P* < 0.05, ** *P* < 0.01

The tissue concentrations of three representative proinflammatory cytokines, TNF-α, IL-8, and IL-1β, were determined. These cytokines are considered to be induced by macrophage infiltration, and they were significantly higher in the prostatitis group. Oral administration of tadalafil markedly suppressed the concentrations of all three cytokines in the NBP rat prostate (*P* < 0.01, *P* < 0.05, *P* < 0.005, respectively; Fig. 4). There was no significant effect of tadalafil on the concentrations of these cytokines in non-prostatitis rats.

## Discussion

Chronic inflammation (mostly nonbacterial and asymptomatic) is associated with symptom development in BPH [4]. To investigate the underlying mechanisms of chronic inflammation in the prostate that are responsible for symptomatic BPH, experimental animal models with inflammatory prostatitis are needed. Kamijo et al. [13] and our group [14–16] used 10-month-old rats treated with 17β-estradiol for 30 days after castration to investigate the effects of therapeutic agents such as cernitin pollen extract and Eviprostat. Therefore, this was an appropriate model to use for evaluation of the effects of drugs on inflammatory changes in the prostate. Although this animal model does not match human BPH in prostate size or glandular hyperplasia, it demonstrates certain characteristics, including severe acinar gland atrophy and stromal predominance, in addition to inflammatory infiltrates, which mimic symptom progression in BPH.

Recently Yamaguchi et al. [12] found that an increase in pelvic pain is induced in this NBP model and that this pain is suppressed by repeated treatment with tadalafil. However, they did not investigate the effects of tadalafil on inflammatory changes. In the present study, we demonstrated for the first time that the administration of tadalafil showed a reduction in macrophage levels and the extent of T-cell infiltration in the same NBP model. Meanwhile, the increased concentrations of TNF-α, IL-8, and IL-1β in the prostate tissue of NBP rats were also suppressed in the tadalafil administration group. We assume that the attenuation of pelvic pain shown by Yamaguchi et al. [12] is partly due to these effects of tadalafil. IL-1β and TNF-α are both pro-inflammatory cytokines and reported to be up-regulated in prostatic sections of prostatitis, suggesting that these are the key markers in inflammatory prostate [17]. IL-8 also seems to be a key mediator in human BPH: its concentrations in prostatic secretions from patients with BPH accompanied by inflammation are higher than in patients with BPH alone [18].

This study is consistent with other available evidence that PDE5-Is reduce chronic inflammation in the prostate, because several investigators have reported their anti-inflammatory effects in different animal models such as metabolic syndrome-induced prostatitis in the rabbit [11] and spontaneously hypertensive rats [10], as well as in human BPH cells [19]. In addition, tadalafil has protective effects on prostatic structural and functional changes induced by chronic pelvic ischemia [20]. Moreover, in a rat model of experimental autoimmune prostatitis, tadalafil reduces prostatic inflammation [21].

The latest evidence on the pathophysiology of LUTS/BPH has provided the rationale for use of PDE5-Is, which bring about improvement of LUT oxygenation, smooth muscle relaxation, negative regulation of proliferation, transdifferentiation of LUT stroma, and reduction of bladder afferent nerve activity [22]. Besides protection of the prostate by tadalafil, several investigators have shown the effect of tadalafil on chronic ischemia-related bladder dysfunction in animal models, including a rat model of chronic pelvic ischemia [23] and bladder outlet obstruction [24]. It has been hypothesized that PDE5-Is could reduce inflammation and the associated fibrosis and improve oxygenation in the human prostate and bladder, with normalization of prostatic and bladder structural anatomy and physiologic activity. All of these effects of tadalafil may contribute to the amelioration of bladder dysfunction.

Previous studies have evaluated the pharmacological effects of tadalafil in animal models, almost all of which used oral administration of tadalafil at doses of 2 or 10 mg/kg. For example, Morelli et al. [10] demonstrated an improvement in prostate gland oxygenation by tadalafil at 2 mg/kg/day for 1, 7 or 28 days. Nomiya et al. [23] have described the protective effects of tadalafil on bladder function at 2 mg/kg/day for 8 weeks in a rat model of chronic bladder ischemia induced by iliac arterial endothelial injury. Kawai et al. [24] reported that the repeated administration of tadalafil at 10 mg/kg/day for 14 days ameliorated the bladder function in a rat model of bladder outlet obstruction. According to these reports, in this study we used tadalafil at 10 mg/kg/day for 30 days. Recently Yoshinaga et al. [25] reported that C_max_ values of 68.5 and 460 ng/ml in rats after single oral doses of tadalafil of 2 and 10 mg/kg, respectively are physiologically relevant with the clinical plasma concentration of tadalafil is 95.6 ng/ml when the drug is administered at 5 mg/person (for BPH patients) and 446 ng/ml when it is administered at 40 mg/person (for PAH patients). Since the doses of tadalafil used in current studies are similar to the plasma concentrations in humans being treated for BPH and PAH, the results we showed in the paper partly may explain the protective mechanisms of Tadalafil in BPH patients.

In the present study, the anti-inflammatory effect of tadalafil was demonstrated in the NBP rat model. However, this model is hormone-induced, and there is are anatomic differences between the rat and the human prostate. The effect of tadalafil on the relief of LUTS cannot therefore be assessed in this model. Despite this experimental limitation, the results obtained in the present study partially clarify the mechanism by which tadalafil prevents the development of BPH through the suppression of nonbacterial inflammation. To elucidate the mechanism of the effect of tadalafil in more detail, further study is needed.

## Conclusion

The phosphodiesterase 5 inhibitor tadalafil suppressed the increase in inflammatory factors and stromal proliferation in a rat model of nonbacterial prostatitis, providing evidence for anti-inflammatory effects of tadalafil in this model.

## Data Availability

The datasets used or analyzed in this study are available from the corresponding author on reasonable request. All data analyzed in this study are publicly available.

## Abbreviations

BPH: Benign prostatic hyperplasia
BW: Body weights
control-tadalafil: control treated with tadalafil
IL-1β: Interleukin-1β
IL-8: Interleukin-8
LUTS: Lower urinary tract symptoms
MTOPS: Medical Therapy of Prostate Symptoms
NBP: Nonbacterial prostatitis
NBP-tadalafil: NBP with tadalafil treatment
NO/cGMP: Nitric oxide cyclic-guanine monophosphate
PDE5-I: Phosphodiesterase type 5 inhibitor
PW: Prostate weights
S/E: Stroma-to-epithelium
TNF-α: Tumor necrosis factor-α
